# A Primer on Multimodal Imaging and Cardiology-Radiology Congenital Heart Interface

**DOI:** 10.3390/children6040061

**Published:** 2019-04-23

**Authors:** Monesha Gupta-Malhotra, William Schaaf, Shelby Kutty

**Affiliations:** 1 Department of Pediatric Cardiology, Johns Hopkins All Children’s Hospital, Johns Hopkins University, Saint Petersburg, FL 33701, USA; 2 Department of Pediatric Radiology, Johns Hopkins All Children’s Hospital, St. Petersburg, FL 33701, USA; wschaaf1@jhmi.edu; 3 Department of Pediatric Cardiology, Johns Hopkins Children’s Center, Baltimore, MD 21205, USA; skutty1@jhmi.edu

**Keywords:** congenital heart disease, multimodality imaging, quality assurance

## Abstract

Pediatric cardiology imaging laboratories in the present day have several modalities for imaging of congenital and acquired cardiovascular disease. These modalities include echocardiography, cardiovascular magnetic resonance imaging, cardiac computed tomography and nuclear imaging. The utility and limitations of multimodal imaging is described herein along with a framework for establishing a cardiology-radiology interface.

## 1. Introduction

Modern cardiac imaging is needed to precisely define the anatomy and physiology of pediatric and congenital heart disease, sometimes requiring multiple modalities [1,2,3]. Newer modalities, including cardiac magnetic resonance imaging (CMR) [4] and cardiac computed tomography angiogram (CCTA) [5,6] are increasingly utilized. Echocardiography [7,8], remains the mainstay of non-invasive diagnosis whereby intervention and surgery for congenital or acquired heart disease are decided upon. More recently, cardiac 3D printing has been shown to be useful for surgical and interventional planning, as well as education of healthcare professionals [9]. Advancements in hardware and software for imaging are rapidly emerging across all these modalities, so ongoing education is necessary to keep up with changing techniques and practice. As these tools can be expensive, judicious use could reduce costs and save time. Understanding the relative strengths and weaknesses of each modality are important for optimal selection in a patient with the goal of providing personalized care.

Integrated multimodal imaging for congenital heart disease in children and adults have been proposed [1,10,11,12]. Comprehensive protocols for individual congenital heart diseases including tetralogy of Fallot [13], transposition of great arteries [14], coronary artery anomalies [15], pulmonary hypertension secondary to congenital heart disease [16], pediatric heart transplantation [17] and others are now available to improve standardization and optimize patient care.

## 2. Types of Noninvasive Multimodality Cardiovascular Imaging

Each imaging modality has disadvantages and advantages as well as environments and indications for which they are ideally suited. The various modalities are briefly described below.

**Figure 1:**
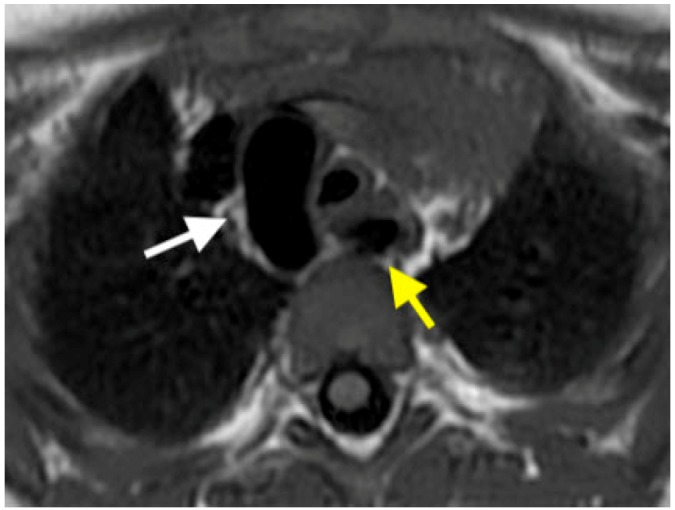
Axial black blood. Cardiac Magnetic Resonance (CMR) image demonstrates a right aortic arch with an aberrant left subclavian artery (arrows).

**Figure 2:**
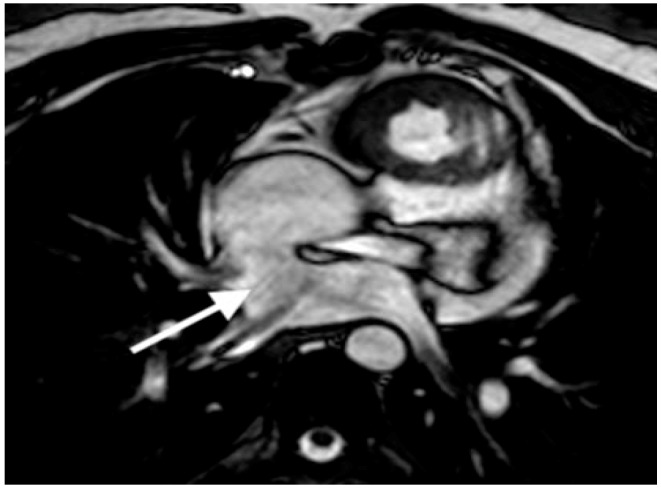
Axial white blood CMR image demonstrates the pulmonary venous baffle (arrow) after Senning operation for D-transposition of the great arteries.

**Figure 3:**
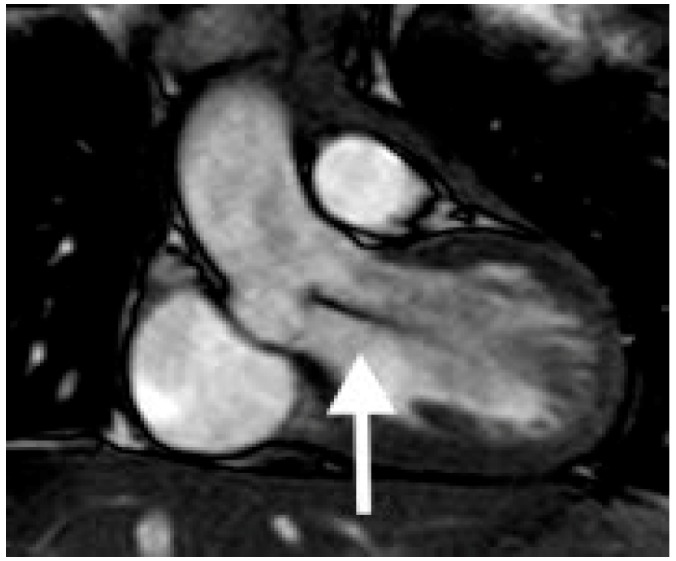
Coronal white blood CMR image demonstrates a dephasing jet (arrow) from aortic valve insufficiency.

**Figure 4:**
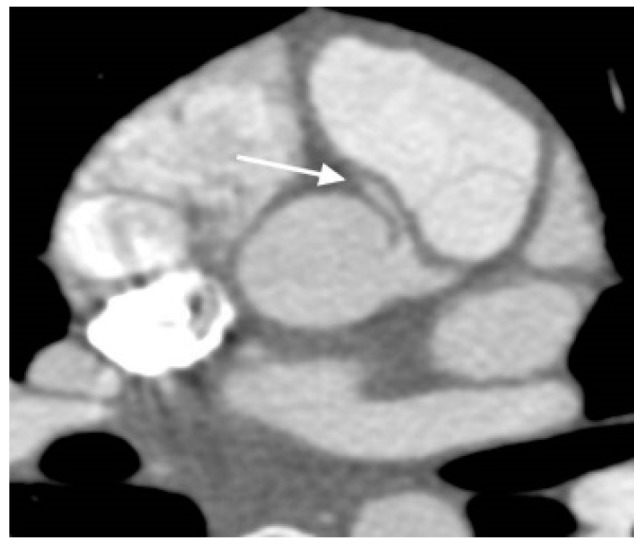
Axial Cardiac Computed Tomography Angiography (CCTA) image demonstrates anomalous right coronary artery origin from the left sinus of Valsalva with intra-arterial course (arrow).

**Figure 5:**
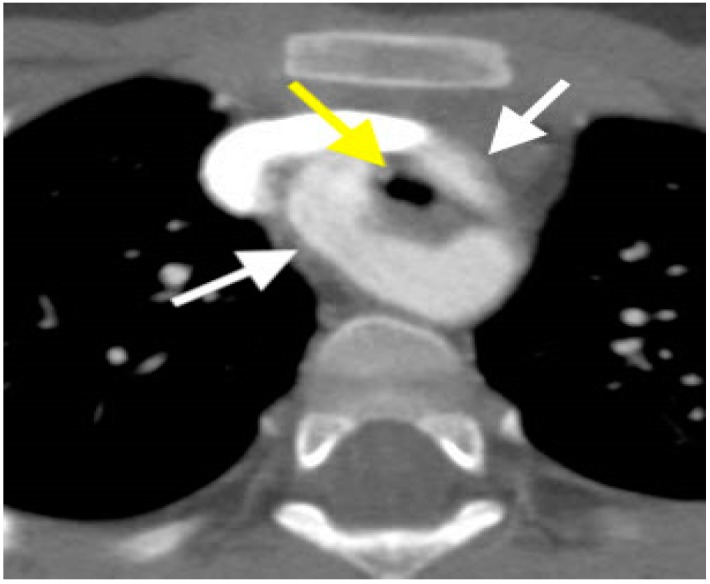
Axial CCTA image showing right dominate double aortic arch (arrows) with tracheal narrowing (arrow on the central dark circle).

**Figure 6:**
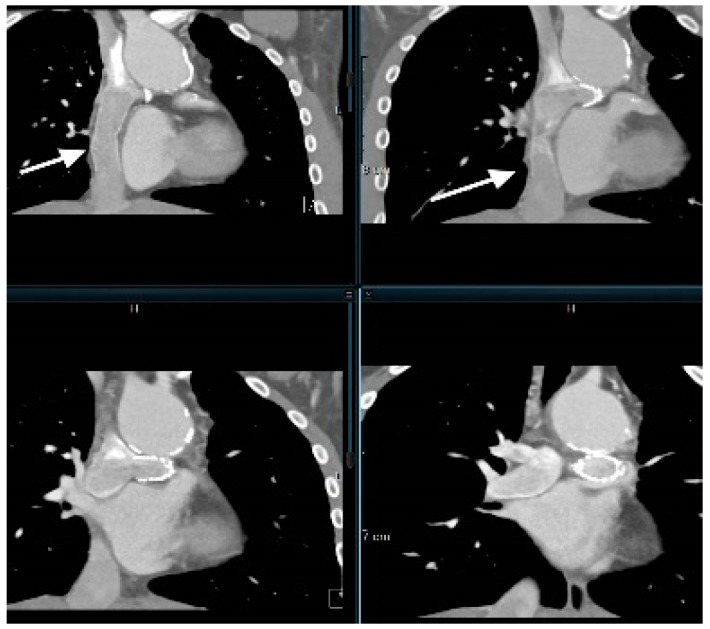
Coronal CCTA image of a cavopulmonary shunt (arrows) in patient with single ventricle congenital heart disease.

- **Echocardiography:** Echocardiography is the ideal imaging modality in the pediatric setting and is the most widely used (Table 1). Beyond standard transthoracic echocardiography, fetal echocardiography [18], transesophageal echocardiography [19], intravascular ultrasound, and intracardiac echocardiography are used.
- **Cardiac Magnetic Resonance Imaging (CMR):** CMR is an adjunct to echocardiography (Table 2) and utilized extensively for imaging congenital heart disease, before (Figure 1) and after surgery, (Figure 2 and Figure 3) [20] and in adults [21]. Evaluating the right ventricle using echocardiography becomes particularly difficult in the adult age [20,22]. CMR has become the reference standard to quantify right ventricular volumes [20,22]. CMR is superior to echocardiography for imaging extracardiac thoracic vasculature, and also for diagnosing myocardial diseases including inflammation, ischemia, scar, and infiltration. It is also an effective tool in the diagnostic work-up of all forms of cardiomyopathies, myocarditis and coronary anomalies (Table 3). However, CMR requires dedicated and trained personnel for image acquisition and interpretration for the best results. It is currently also limited in the assessment of diastolic function. The use of contrast with CMR is limited by patient’s renal function. Children younger than 7 years of age often need general anesthesia for CMR. The limitations of CMR in children with implanted devices are decreasing with newer CMR compatible devices. Finally, stress CMR could potentially be useful in the assessment and risk stratification of congenital or acquired coronary artery disease. CMR compatible catheterization laboratories are emerging, which can reduce the lifelong risk for radiation exposures in patients with congenital heart disease [23].
- **Cardiac Computed Tomography Angiography (CCTA):** The CCTA is valuable for diagnostic assessment of congenital heart disease (Table 4). It causes substantially less radiation exposure compared to cardiac catheterization and can be optimized to very low radiation dosing in moderan scanners [24]. It can also make rapid anatomic assessment of the entire body, in particular the lungs. CCTA has important role in very ill patients, small patients, or in complex anatomy. It is particularly helpful for the assessment of coronary arteries (Figure 4), and extracardiac structures (Figure 5 and Figure 6) including aortic arch, pulmonary veins and pulmonary arteries, as it has one of the best spatial resolutions [25]. CCTA takes much less time and anesthesia than a CMR, and can be performed in the presence of ferromagnetic devices. Other indications include left atrial scar mapping prior to a maze procedure with pulmonary vein isolation, assessment of in-stent luminal stenosis due to intimal hyperplasia, and work-up of athletes for cardiomyopathies (Table 5). The rapid evolution of technology and the ability to make submillimeter volumetric datasets generates exceptionally good quality images at high speed and resolution. Furthermore, computer-assisted calculation of differential lung and blood volumes can be performed.
- **Nuclear Imaging:** Nuclear imaging of the heart and other organs uses a radiopharmaceutical agent such as thallium-201 and a detection device such as a gamma camera, positron camera, or rectilinear scanner. Clinical applications of cardiac radionuclide imaging are the gated cardiac blood pool scan, myocardial imaging, and detection of myocardial necrosis. Radiation exposure is a significant limitation.

## 3. Multimodality Imaging Laboratory and Quality Assurance

Integration of imaging services is necessary to accommodate the growing imaging needs of congenital heart programs and to provide value at reasonable cost. Programs should coordinate multimodal imaging services by sharing and developing tools that can engage physicians and imaging personnel in the shared responsibility for judicious use. The cost effectiveness of multimodal imaging is being evaluated by third party payers in the United States [26]. Payments and reimbursements are in a state of flux and may change further over time. It is essential to continually assess patterns of care in an effort to understand and improve the rate of clinically appropriate imaging (high-value imaging), while reducing inappropriate testing. Integration of imaging modalities require attention to several domains of quality, some of which are listed below:

- **Quality Control and Safety:** The following aspects are required: (a) Setting standards to ensure the reliability and technical quality of diagnostic images produced; (b) Processes to ensure that equipment and software meet performance specifications; (c) Standards and processes in place to ensure the safety of imaging providers and patients, (d) Certification or accreditation as an independent and transparent evaluation of imaging facilities, and for laboratories to demonstrate accountability and high standards, and (e) Development of mock drills with a check list for all core personnel.
- **Appropriate Use Criteria (AUC):** AUC defines when and how often it is reasonable to perform a given procedure or test [27]. An appropriate imaging test is one in which the expected incremental information, combined with clinical judgment, exceeds the expected negative consequences by a sufficiently wide margin for a specific indication that the procedure is generally considered acceptable care and a reasonable approach for the indication [27]. Multiple criteria are required to determine AUC, including review of the clinical data, knowledge of the diagnostic tests and procedures, past clinical experiences, and the availability of equipment and/or personnel.
- **Layering of Tests:** Multiple tests in the same patient can lead to additional costs and lengthy work-flow. Therefore, knowledge of various available modalities and application of the appropriate one for the patient is key [26]. This type of approach need to be developed among the treating physicians.
- **Trends in Healthcare and Reimbursement:** The rate of imaging volume growth in Medicare has been slowing since 2005 and imaging spending has dropped significantly from previous years [26]. There are ongoing attempts at both the state and national level to eliminate or limit the ability of cardiologists to provide diagnostic imaging services in their offices. The American College of Cardiology strongly supports the ability of specialty physicians, who have knowledge of specific organ systems and disease states, as well as of their patients’ needs, to provide timely and convenient access to imaging services while curtailing costs.

## 4. Developing a Multimodality Imaging Laboratory

There are several challenges of developing a state of the art advanced imaging program in a pediatric hospital. The available modalities are usually stand-alone services at hospitals, with intermittent referrals to each other creating barriers to integration. Therefore, currently there is misalignment between the availability of imaging modality and its utilization for pediatric heart disease. There are various reasons for this limited integration, including scattered expertise and experience, deficiency of core imaging physicians and technicians who are cross-trained, and some lack of proper equipment.

A major limitation is the high degree of specializations needed for each imaging modality [28]. For example, CMR and CCTA requires specializations in pediatric cardiology and radiology, and cross training for competency. Also, the available imaging modalities are rapidly undergoing technological advances that one has to stay updated of. If a “state of the art” imaging laboratory is envisioned, then the team must be committed to collaboratively bridge any gaps.

## 5. Cardiology-Radiology Interface

Personnel with different specializations are required to bring together the multimodal aspect of imaging to the cutting-edge potential, reduce the costs and improve patient care. Having a team approach, standardization and protocolization helps integrate the different modalities. Collaboration should be viewed as a collective gain, and the focus should always be on the patient in order to succeed. Pediatric imaging labs should work as a team towards:

- Coordinating multimodal imaging services and integrating them to optimize imaging. For example, cardiac 3D and 4D modeling from advanced imaging could be valuable in complex congenital heart disease [29].
- Identifying a cadre of physicians with a congenital heart focus who will regularly attend education programs to share ideas, collaborate and acquire knowledge. The core team members from various modalities with congenital heart focus must be identified and brought together. In that way fresh ideas to improve quality and outcomes would emerge. Team members should be available after hours for emergent imaging on a call schedule, and effectively communicate with the referring providers.
- Developing protocols for image acquisition. The team members should develop, implement and assist in maintaining the required knowledge for protocols and techniques.
- Developing protocols for congenital heart disease reporting using a segmental approach. In this way lesions are not missed on routine studies.
- Delivery of high-quality, post-processed images. Providing clinically relevant visualization and analysis of medical imaging data to patient families and health care providers requesting such services.
- Optimizing image guided interventions in laboratories. Currently transesophageal echocardiogram is routinely used for this purpose in the cardiac catheterization laboratory and in the operating room. CMR using non-ferromagnetic equipment is being used in select laboratories.
- Designing quality measures and applying AUC [26] with a goal to share and develop tools that can engage all stakeholders in the shared responsibility of judicious use of imaging services and reducing radiation dose [30].

## 6. Avenues for Collaboration and Teaching

The education and research mission of the cardiovascular imaging lab is paramount for advancement in the field. Knowledge precedes imaging, and correct understanding leads to correct imaging and, thus, the diagnosis. The aims should include developing and applying new, advanced visualization techniques toward that purpose. The team should also work towards developing didactic courses and lecturers and run hands-on workshops. Furthering education with an aim towards, but not limited to, the following should be considered:

(a) Partnering with referring providers: There should be written guidelines in every imaging laboratory which a referring medical provider can use for obtaining an advanced imaging test for their patients.Partnering with ordering clinicians can help reduce inappropriate testing. Electronic Medical Records (EMR) reporting systems should be in compliance with The Intersocietal Commission for the Accreditation of Echocardiography Laboratories (ICAEL), American College of Radiology and so on, as well as understandable to the referring physicians.
(b) Pediatric Cardiology/Adult Cardiology/Radiology/Maternal Fetal Medicine/Emergency Room Ultrasound Trainees: Developing a didactic lecture series for trainees on multimodal imaging. It is important to provide assistance and mentorship to faculty and trainees, including training on new techniques or procedures, research and publications.
(c) Developing a Non-Invasive Cardiac Imaging Fourth-Year Fellowship: Goal of the fellowship is to provide advanced training opportunity in non-invasive cardiac imaging the intent of preparing the advanced trainee for a future faculty position within an academic or a non-academic setting and independently run an imaging lab.

## 7. Conclusions

Multimodality imaging is necessary for ascertaining all aspects of a particular congenital heart disease in order to tailor the therapeutic approach, including electrophysiological, interventional and surgical. A team effort is required to establish the best approach and outcome in a congenital heart disease.

## Figures and Tables

**Table 1:** Echocardiography.

**Advantages of Echocardiography:**
a. Portability
b. Non-invasive
c. No radiation
d. Excellent temporal resolution
e. Good spatial and contrast resolution
f. Invaluable in rapid hemodynamic assessment
g. Most suitable for valve anatomy and function
**Limitations of Echocardiography:**
a. Limited in its ability to visualize extracardiac structures
b. Poor spatial resolution with limited acoustic windows in the setting of obesity, or after surgery
c. Less accurate for cardiomyopathies, acute myocarditis and ischemia
d. Less accurate for coronary artery anomalies
e. Less accurate quantification of volumes and masses
f. Doppler angle dependence for quantification

**Table 2:** Cardiac magnetic resonance imaging.

**Advantages of CMR over Cardiac Catheterization for Congenital Heart Disease Imaging:**
(1) Non-invasive
(2) It requires shorter sedation times
(3) It does not expose the patient to ionizing radiation
(4) There is excellent spatial and contrast resolution
**Advantages of CMR over CTA in Imaging Congenital Heart Disease Are:**
1. It does not expose the patient to ionizing radiation
2. CMR can image flowing blood without intravenous contrast

**Table 3:** Indications for cardiac magnetic resonance imaging in pediatric cardiology.

1. Congenital heart defects
2. Myocarditis
3. Coronary artery anomalies/Kawasaki disease
4. Extracardiac anatomy in heterotaxy syndromes
5. Pulmonary venous and arterial assessment
6. Pericardial disease
7. Cardiac tumor/mass
8. Fabry’s disease during enzyme replacement
9. Cardiomyopathy (hypertrophic, dilated, restrictive and arrhythmogenic dysplasia)
10. Ventricular function and ventricular mass
11. Aortopathy
12. Cardiac stress perfusion study

**Table 4:** Cardiac computed tomography angiogram.

**Advantages of CCTA over Cardiac Catheterization in Imaging Congenital Heart Disease Are:**
1. Less invasive
2. Faster
3. Requires much shorter sedation times, if at all
4. Exposes the patient to lower doses of ionizing radiation
5. Not limited in the field of view as it is a 3-d model (angiography is a 2-d modality)
6. Not susceptible to difficulties in resolving overlapping arterial and venous flow
**Advantages of CCTA over CMR in Imaging Congenital Heart Disease Are:**
1. Requires much shorter sedation times
2. More easily accessible
3. Has better spatial and temporal resolution
4. Ability to visualize despite use of prosthetic material—vascular stents, coils, occluder devices (unlike artifacts from metallic-induced inhomogeneity in the magnetic field due to significant signal dephasing and signal loss)
5. Availability in an emergency setting for fast imaging with little respiratory motion in potentially unstable patients.

**Table 5:** Indications for Computed Tomography Angiogram in pediatric cardiology.

1. Coronary artery anomalies
2. Kawasaki disease and other vasculitis
3. Congenital heart defect
4. Extracardiac anatomy in heterotaxia
5. Pulmonary venous and arterial assessment
6. Aortopathy
7. Pericardial disease
8. Cardiac thrombus
9. Cardiomyopathy (hypertrophic, dilated, restrictive and ischemic)
10. Extracardiac anatomy in heterotaxy syndromes
11. Pulmonary sequestration to check arterial supply and venous drainage
